# Molecular hydrogen regulates gene expression by modifying the free radical chain reaction-dependent generation of oxidized phospholipid mediators

**DOI:** 10.1038/srep18971

**Published:** 2016-01-07

**Authors:** Katsuya Iuchi, Akemi Imoto, Naomi Kamimura, Kiyomi Nishimaki, Harumi Ichimiya, Takashi Yokota, Shigeo Ohta

**Affiliations:** 1 Department of Biochemistry and Cell Biology, Graduate School of Medicine, Nippon Medical School, 1–396 Kosugi-machi, Nakahara-ku, Kawasaki-city, Kanagawa 211–8533, Japan; 2 Department of Neuroregenerative Medicine, Juntendo University Graduate School of Medicine, 2-1-1 Hongo, Bunkyo-ku, Tokyo 113–8421, Japan

## Abstract

We previously showed that H_2_ acts as a novel antioxidant to protect cells against oxidative stress. Subsequently, numerous studies have indicated the potential applications of H_2_ in therapeutic and preventive medicine. Moreover, H_2_ regulates various signal transduction pathways and the expression of many genes. However, the primary targets of H_2_ in the signal transduction pathways are unknown. Here, we attempted to determine how H_2_ regulates gene expression. In a pure chemical system, H_2_ gas (approximately 1%, v/v) suppressed the autoxidation of linoleic acid that proceeds by a free radical chain reaction, and pure 1-palmitoyl-2-arachidonyl-*sn*-glycero-3-phosphocholine (PAPC), one of the major phospholipids, was autoxidized in the presence or absence of H_2_. H_2_ modified the chemical production of the autoxidized phospholipid species in the cell-free system. Exposure of cultured cells to the H_2_-dependently autoxidized phospholipid species reduced Ca^2+^ signal transduction and mediated the expression of various genes as revealed by comprehensive microarray analysis. In the cultured cells, H_2_ suppressed free radical chain reaction-dependent peroxidation and recovered the increased cellular Ca^2+^, resulting in the regulation of Ca^2+^-dependent gene expression. Thus, H_2_ might regulate gene expression via the Ca^2+^ signal transduction pathway by modifying the free radical-dependent generation of oxidized phospholipid mediators.

Molecular hydrogen (H_2_) was originally thought to behave as an inert gas in mammalian cells; however, our previous studies showed that this is not always the case^1^, demonstrating that H_2_ neutralizes the hydroxyl radical (·OH) and peroxynitrite (ONOO^–^) inside cells and acts as a novel antioxidant to protect the cells against oxidative stress^1^,^2^. Inhalation of 1%–4% (v/v) H_2_ gas is effective for the treatment of ischemia/reperfusion injuries^1^,^3^,^4^. Recently, inhalation of 1.3% H_2_ gas from a premixed gas was shown to protect neurons in a cardiac arrest model^5^. However, the mechanism of how such a low concentration of H_2_ exerts the positive effects is not known.

Numerous studies have strongly suggested that H_2_ has the potential for a variety of therapeutic and preventive applications^6^,^7^. In addition to extensive animal experiments, more than 10 clinical studies examining the efficacy of H_2_ have been reported^6^,^7^, including double-blinded clinical studies in patients with Parkinson’s disease and rheumatism^8^,^9^. Based on these studies, the field of hydrogen medicine is rapidly growing.

Subsequently, H_2_ was shown to exhibit multiple functions, including anti-inflammatory, anti-apoptotic, anti-allergic, and antioxidant activities, as well as regulation of differentiation and energy metabolism^6^,^7^. To exert multiple functions in addition to anti-oxidative roles, H_2_ regulates various signal transduction pathways and the expression of many genes^6^,^7^. For examples, H_2_ protects neural cells and stimulates energy metabolism by stimulating the hormonal expression of ghrelin^10^ and fibroblast growth factor 21 (FGF21)^11^, respectively. In contrast, H_2_ relieves inflammation by decreasing pro-inflammatory cytokines^12^. However, it is difficult to explain the molecular mechanisms by which H_2_ exerts these functions by conventional concepts alone. To understand the molecular mechanisms by which H_2_ exerts these multiple functions, it is essential to identify the primary targets of H_2_ that modulate signal transduction and gene expression.

Therefore, in this study, we aimed to elucidate one of the molecular mechanisms by which H_2_ mediates signal transduction and gene expression. Our results suggested that low concentrations of H_2_ modulated Ca^2+^ signal transduction and regulated gene expression by modifying the production of oxidized phospholipid species.

## Results

### H_2_ accumulated in the lipid phases

**Figure 1:**
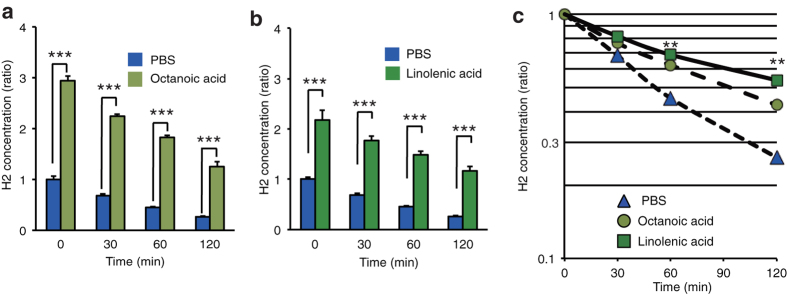
Solubility of H_2_ in fatty acids in the presence of an aqueous solvent. H_2_-saturated phosphate-buffered saline (PBS) was mixed with the same volume (75 mL) of saturated fatty acid (octanoic acid) (**a**) or unsaturated fatty acid (linolenic acid) (**b**) and maintained for 16 h in a closed aluminum bag as described in Methods. The same volume (3 mL) of each phase was transferred to each open tube (ϕ13 mm), followed by measurement of H_2_ at the indicated time (*n* = 4). The experiments were performed at 25 °C. ***P < 0.001 *vs*. PBS. (**a**,**b**) Significance was calculated using an unpaired two-tailed Student’s t-test. (**c**) Time courses of retention times of H_2_ in each phase in the open vessels. **P < 0.01, *vs*. octanoic acid (*n* = 4).

To understand the difference between intracellular conditions and aqueous solutions, we focused on the lipid phases to determine the intracellular localization of H_2_ accumulation at room temperature. H_2_ incorporation was two- or three-fold higher in the liquid fatty acid phases than in the aqueous phase in the presence of both water and fatty acids, and was retained longer in the fatty acid phases than in the aqueous phase in open vessels (Fig. 1a,b). In particular, H_2_ seemed to be retained significantly longer in the unsaturated fatty acid (linolenic acid) than in the saturated fatty acids (octanoic acid) (Fig. 1c), although this difference in retention time might be attributed to the difference in the number of carbons. Since unsaturated fatty acids are the primary targets for initiating a free radical chain reaction, we assumed that H_2_ could efficiently suppress this reaction in biomembranes, even at low concentrations.

### Autoxidation of unsaturated fatty acids was suppressed by low concentrations of H_2_ gas

**Figure 2:**
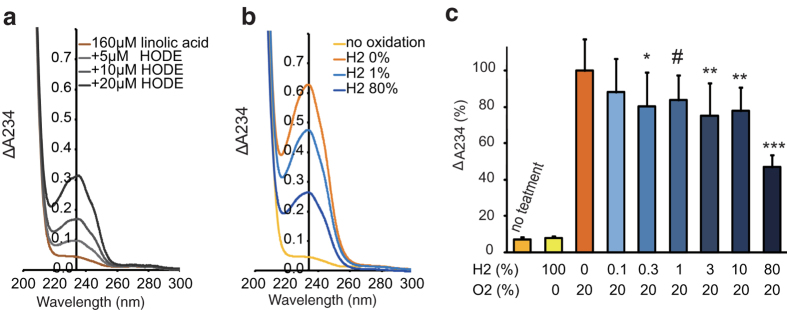
Suppression of autoxidation of linoleic acid-film by H_2_ gas. (**a**) Profile of ultraviolet absorption of 9-hydroxyoctadecadienoic acid; CH_3_(CH_2_)_4_-CH = CH-CH = CH-CH(-OH)-(CH_2_)_7_-COOH) (9-HODE) in cyclohexane, shown as a standard conjugated diene. (**b**) Linoleic acid-film was autoxidized at 37 °C for 20 h in a glass tube placed in a closed aluminum bag in the presence of various concentrations of H_2_ and O_2_ as described in Methods. Representative profiles of ultraviolet absorption of the cyclohexane solution of H_2_-dependent autoxidized linoleic acid are shown. (**c**) Linoleic acids autoxidized with various concentrations of H_2_ were evaluated by measuring absorption at 234 nm. *P = 0.034 (0.3% H_2_), #*P* = 0.069 (1% H_2_), **P < 0.01 (3% H_2_, 10% H_2_), and ***P < 0.001 (80% H_2_) *vs*. 0% H_2_ (*n* = 15).

Autoxidation of unsaturated fatty acids proceeds by a free radical chain reaction in air^13^. Thus, we measured autoxidation of a filmy di-unsaturated fatty acid (linoleic acid: R-CH = CH-CH_2_-CH = C-R’) at 37 °C for 20 h in the dark in the presence of various concentrations of H_2_ gas. A conjugated diene, which should be formed by autoxidation, was estimated by the absorption at 234 nm (Fig. 2a). The absorption at 234 nm was increased depending on the formation of the conjugated diene [R-CH = CH-CH = CH-CH(-OOH)-R’ or R-CH(-OOH)-CH = CH-CH = CH-R’] accompanied by peroxidation in a pure chemical system (H_2_, O_2_, and N_2_ were supplied from gas cylinders) (Fig. 2b). O_2_ was essential for autoxidation (Fig. 2c). As a result, even only approximately 1% H_2_ gas significantly suppressed autoxidation of linoleic acid at 37 °C, even in the absence of any catalysts in the dark in a pure chemical system (Fig. 2c).

### Ca^2+^ signal transduction by H_2_-dependent chemical oxidation of phospholipids

Phospholipids are converted into oxidized mediators that modulate various signal transduction pathways by not only enzymatic reactions, but also by chemical oxidation^14^,^15^. Oxidized phospholipids, including 1-palmitoyl-2-(5-oxovaleroyl)-*sn*-glycero-3-phosphocholine (POVPC) and 1-palmitoyl-2-glutaroyl-*sn*-glycero-3-phosphocholine (PGPC), are present in oxidatively modified low-density lipoproteins (oxLDLs) and have been found in atherosclerotic lesions^15^. These compounds are important as inducers of different cellular responses, including inflammation, proliferation, and cell death. Moreover, autoxidation of 1-palmitoyl-2-arachidonyl-*sn*-glycero-3-phosphocholine (PAPC) leads to the chemical production of various bioactive phospholipid species, such as POVPC, PGPC, 1-palmitoyl-2-(5-hydroxy-8-oxooct-6-enoyl)-*sn*-glycero-3-phosphocholine (HOOA-PC), and 5-hydroxy-8-oxo-6-octenedioic acid (HOdiA-PC)^14^,^15^.

**Figure 3:**
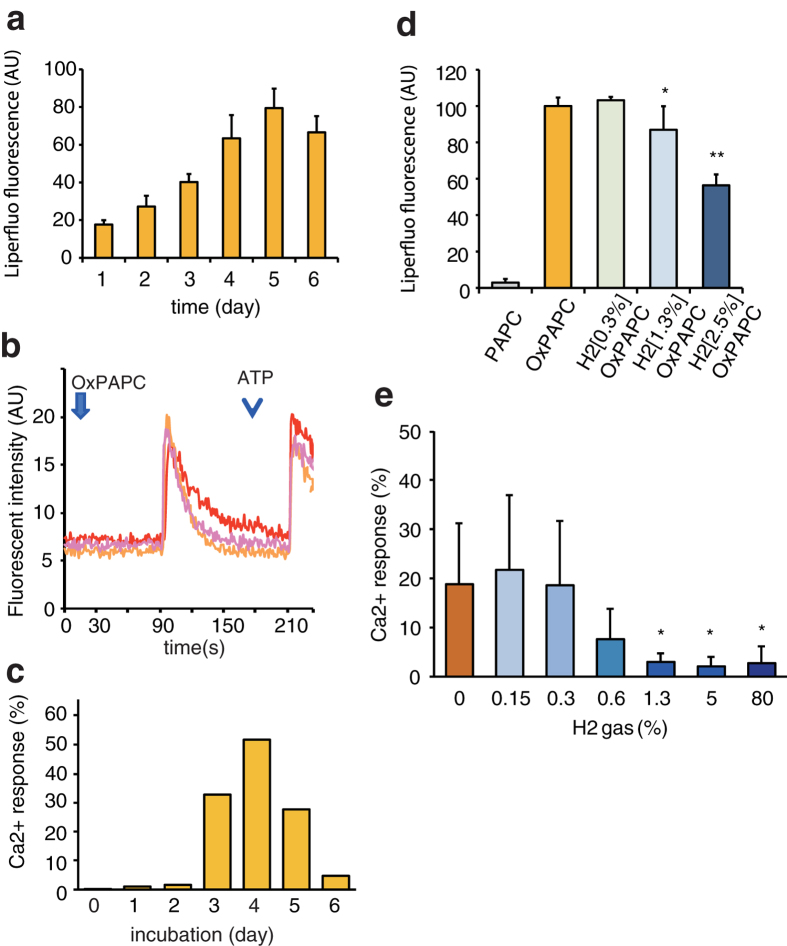
PAPC autoxidized with H_2_ modulated Ca^2+^ signaling. (**a**) Chemically pure PAPC was autoxidized in air with 100% humidity at 25 °C in a closed aluminum bag for the indicated periods, and time-dependent production of peroxides in air from chemically pure PAPC was estimated using Liperfluo fluorescence, where wavelengths of excitation and emission were set at 488 and 535 nm, respectively, as described in Methods. (**b**) Representative responses in THP-1 by OxPAPC with the fluorescent Ca^2+^ indicator Fluo4-AM are shown as described in Methods. The arrow and arrowhead indicate the addition of OxPAPC and ATP, respectively. ATP (a ligand of the Ca^2+^ channel P2X7) was used as a positive control. (**c**) PAPC was autoxidized for the indicated periods at 25 °C, and subjected to the Ca^2+^-signaling assay in THP-1 cells. The OxPAPC-induced Ca^2+^ response depended on autoxidizing period of OxPAPC. (**d**) PAPC was autoxidized in air for 3 days in the absence or presence of the indicated concentrations of H_2_ (H_2_OxPAPC), and the peroxide of OxPAPC or H_2_OxPAPC was estimated using Liperfluo as described in (**a**) (*n* = 3-6). *P = 0.044, **P < 0.01. (**e**) PAPC was autoxidized in air for 3 days with the indicated concentrations of H_2_ (H_2_OxPAPC) and then subjected to Ca^2+^ signaling assays as described in Methods (*n* = 6). *P = 0.021 (1.3%H_2_), *P = 0.022 (5% H_2_), and *P = 0.030 (80% H_2_) *vs*. no H_2_.

We assumed that low concentrations of H_2_ would influence some chemical reactions leading to the production of putative oxidized lipid mediators for the modulation of signal transduction. Because PAPC is one of the major phospholipids in mammalian biomembranes, the role of H_2_ in the chemical production of oxidized phospholipid mediators was determined by conducting autoxidation of pure PAPC (resulting in OxPAPC) in the absence of any catalysts in the dark. The peroxidation of PAPC in air was confirmed by an increase in the signal for the fluorescent dye specific to lipid peroxides, Liperfluo (Fig. 3a). A previous study indicated that OxPAPC activates transcription factors involved in Ca^2+^ signaling^16^. Indeed, when THP-1 cells (a human monocytic cell line derived from a patient with acute monocytic leukemia) were exposed to OxPAPC, a transient increase in cellular Ca^2+^ was observed when a Ca^2+^-sensitive fluorescent dye, Fluo4-AM was used (Fig. 3b). This Ca^2+^ signaling depended on OxPAPC in an oxidation time-dependent manner (Fig. 3c).

Next, the H_2_-dependent production of OxPAPC, which leads to the activation of Ca^2+^ signaling, was investigated by autoxidizing PAPC for 3 days at 25 °C in air at various concentrations of H_2_ (designated as H_2_OxPAPC, and the notation of H_2_[x%]OxPAPC was used when autoxidized in the presence of x% H_2_). H_2_ suppressed the generation of total peroxides as revealed by Liperfluro fluorescence intensity (Fig. 3d). Ca^2+^ signaling was observed when PAPC was autoxidized with less than 0.3% H_2_, whereas more than 1.3% H_2_ significantly disrupted this signaling (Fig. 3e).

In order to investigate the molecule(s) influenced by H_2_, we analyzed H_2_OxPAPCs by using mass spectrometry on autoxidation day 3. In all, 209 bands were detected, with molecular masses ranging from 126.3754 to 991.6494 Da; this was consistent with the findings of a previous report^15^ (Supplementary Fig. 1). The differences in the production of H_2_OxPAPC and OxPAPC species were presented using a heat map (Supplementary Fig. 1i). The levels of many bands were increased or decreased with differences in concentrations of H_2_. For examples as the relatively increased species, the levels of the Ca^2+^ signaling inducers POVPC^16^, HOOA-PC, HOdiA-PC, and hydroxyeicosatetraenoic acid-3-phosphocholine (HETE-PC)^17^ were slightly increased in response to H_2_ (Supplementary Fig. 1i).

Because the reduced form of POVPC was reported to function as an antagonist^18^, it is possible that increased levels of the reduced form(s) of some OxPAPC species, rather than the decreased levels of putative agonists (such as POVPC), might have disrupted Ca^2+^ signaling as a putative antagonist(s). Further studies are warranted to identify the H_2_-dependent bioactive mediator(s).

### Comprehensive analysis of H_2_-dependent regulation of gene expression

**Figure 4:**
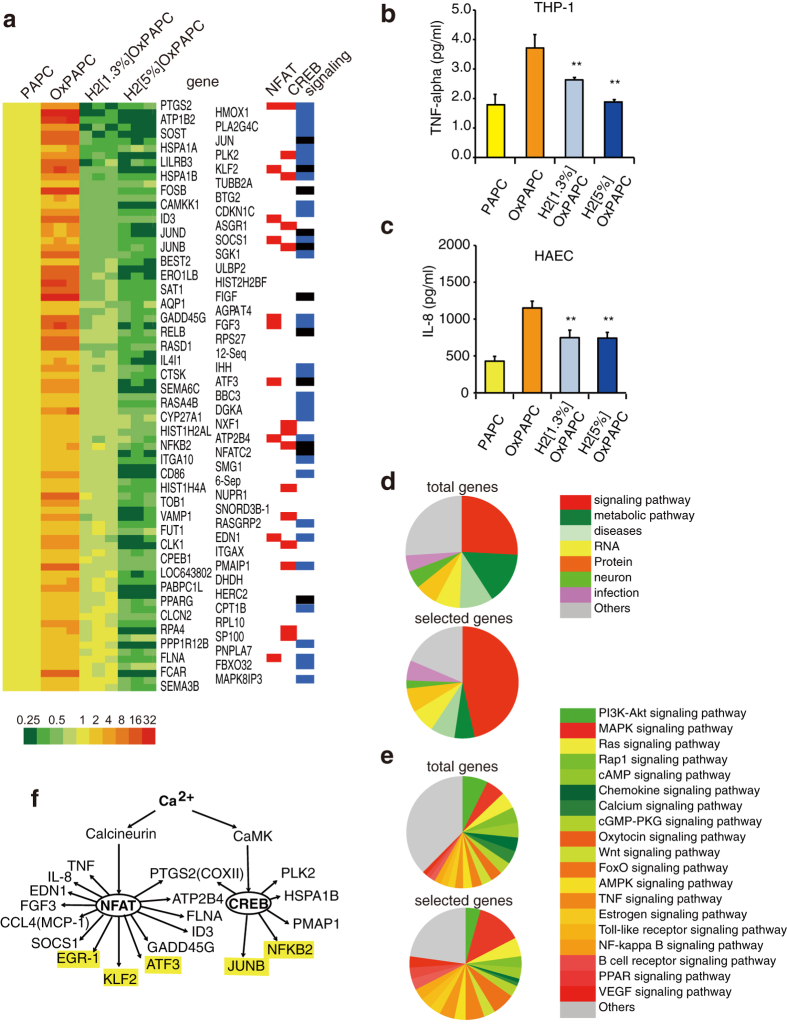
Changes in gene expression regulated by H_2_OxPAPC. (**a**) Three samples of PAPC, OxPAPC, and H_2_OxPAPC were exposed to THP-1 cells for 4 h, and the gene expression was comprehensively analyzed using microarray. Eighty-six genes were selected according to the following criteria; genes up-regulated by OxPAPC (more than 2.5-fold, *vs.* PAPC) and those down-regulated by H_2_[1.3%]OxPAPC and H_2_[5%]OxPAPC (less than 0.75-fold and 0.5-fold, respectively, *vs*. OxPAPC) are shown in a heat map (red and green indicate the up-regulation *vs.* PAPC treatment, and the down-regulation *vs.* OxPAPC treatment, respectively, as shown in the color gradient). Possible target genes of NFAT and CREB are marked with red on the right. Genes encoding factors involved in signal transduction and transcription are indicated by blue and black, respectively, on the right. The release of TNF-α (**b**) (from THP-1) and IL-8 (**c**) (from HAEC) was investigated using ELISA as described in Methods. (**d**, upper) Ratio of genes belonging to each category for a total of 7,142 genes identified by the KEGG database. (**d**, lower) Ratio of genes belonging to each category in the 86 selected genes listed in a. (**e**, upper) Ratio of genes belonging to each signaling pathway identified by the whole KEGG database. (**c**, lower) Ratio of genes belonging to each signaling pathway in the selected genes listed in (**a)**. (**f**) The H_2_OxPAPC-dependent expression of genes transcribed by CREB and NFAT. Transcription factors are indicated in yellow.

Next, we investigated how H_2_OxPAPC influences gene expression. PAPC was autoxidized in the absence or presence of various concentrations of H_2_ for 3 days and then administered to cultured THP-1 cells. In a preliminary experiment, the change in the expression level of tumor necrosis factor (TNF)-α gene in response to OxPAPC from that to H_2_OxPAPC peaked at 4 h. Thus, by using microarray analysis, we comprehensively analyzed the change in gene expression in response to the H_2_-dependent mediators at 4 h in three samples under each condition. In all, 86 genes were selected according to the following criteria as described in the legend of Fig. 4a: a significant increase in OxPAPC (*vs.* PAPC), and a significant decrease in H_2_[1.3%]OxPAPC and H_2_[5%]OxPAPC (*vs.* OxPAPC) (Supplementary Table 1). The gene expression profile was presented in a heat map (Fig. 4a). The selected genes were validated by semi-quantitative real-time polymerase chain reaction (RT-PCR), and marginal changes in the expression levels of some genes were confirmed (Supplementary Fig. 2).

In addition, the regulatory expression of TNF-α and IL-8 by H_2_OxPAPC was investigated using THP-1 and a different cell type (human aortic endothelial cells: HAEC), respectively (Fig. 4b,c).

According to the Kyoto Encyclopedia of Genes and Genomes (KEGG) Pathway Database (http://www.genome.jp/kegg/pathway.html), the functions of 7,143 genes were identified and classified (Fig. 4d, upper). We classified the 86 selected genes (Fig. 4e, lower). Of these 86 genes, 46.5% belonged to those involved in signaling pathways (Fig. 4d, upper), whereas 25.8% of the total number of 7,143 genes is involved in signaling pathways (Fig. 4d, lower). Genes encoding factors involved in signal transduction and transcription factors are indicated by blue and black, respectively, on the right in the heat map (Fig. 4a).

Among the genes involved in signaling pathways, the proportion of those belonging to Ca^2+^ signaling were lower in the selected genes than in those in the entire genome, indicating that H_2_ regulates fewer components of the Ca^2+^ signaling pathways (Fig. 4e, lower). This was consistent with the finding that H_2_OxPAPC decreased Ca^2+^ signaling. In contrast, the proportion of genes belonging to the mitogen-activate protein kinase (MAPK) signaling was higher (Fig. 4e, lower), indicating that H_2_ regulates more components of MAPK signal transduction pathways (Fig. 4e, lower).

The signal transduction pathways that were regulated by H_2_ are shown in Supplementary Table 1 according to the KEGG Pathway Database. These data suggested the possibility that low concentrations of H_2_ contribute to various signal transduction pathways via oxidized phospholipid species.

cAMP response element binding protein (CREB)-target genes were selected according to the CREB Target Gene Database (http://natural.salk.edu/CREB/), and nuclear factor of activated T cells (NFAT) target genes were selected by referring to Medline, as shown in Supplementary Table 1. The target genes of CREB and NFAT are marked by red on the right in the heat map panel as NFAT or CREB (Fig. 4a). A considerable number of the selected genes were targets of CREB or NFAT (Fig. 4 f). These data are consistent with the findings of previous studies showing the Ca^2+^-dependent regulation by these transcription factors: CREB is activated via phosphorylation by a calmodulin-dependent kinase (CaMK)^19^ in a Ca^2+^-dependent manner, and NFAT is dephosphorylated by calcineurin (CN) in a Ca^2+^-dependent manner, translocates to the nucleus, and then functions as a transcription factor with its partner proteins, e.g., activator protein 1 (AP-1), CREB, or nuclear factor-kappa B (NF-κB)^20^. Indeed, exposure of THP-1 to OxPAPC, but not to H_2_OxPAPC, stimulated the nuclear translocation of NFAT (Supplementary Fig. 3).

Thus, H_2_-dependent oxidized mediators or putative antagonists could be associated with transcriptional regulation via Ca^2+^ signaling.

### Free radical inducers contributed to the NFAT pathway in cultured cells

**Figure 5:**
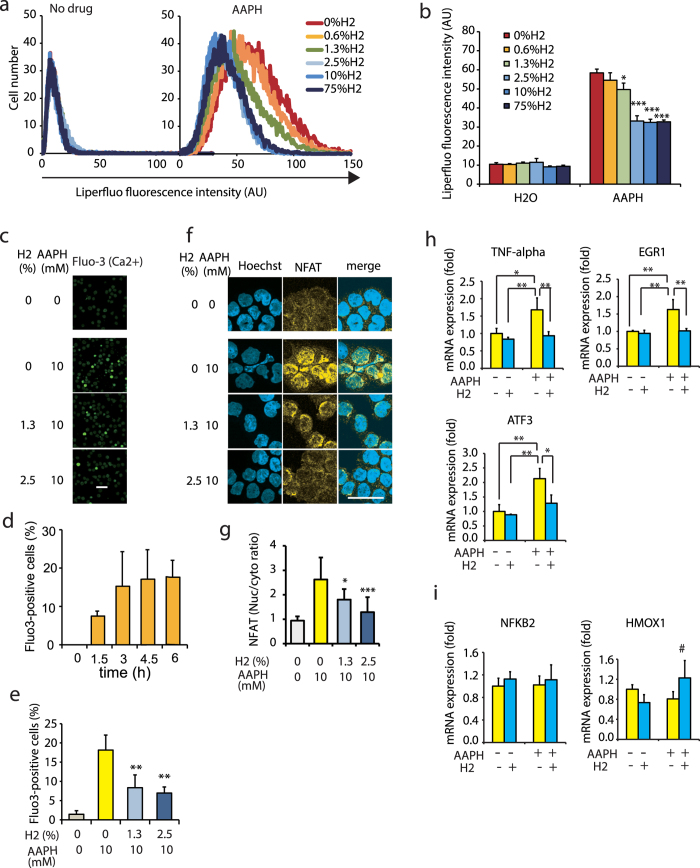
H_2_ suppressed free radical inducer-dependent fatty acid peroxidation and Ca^2+^ and NFAT signaling. (**a**) THP-1 was exposed to a free radical inducer (10 mM AAPH) in the absence or presence of the indicated concentrations of H_2_ for 4.5 h. Representative flow cytometric profiles are shown to demonstrate lipid peroxides with Liperfluo signals. (**b**) The Liperfluo signals were quantified. *P = 0.015, ***P < 0.001 *vs*. 0% H_2_ (*n* = 6). (**c**) THP-1 cells were treated with 10 mM AAPH for 3 h in the presence of the indicated concentrations of H_2_. Intracellular Fluo-3 fluorescence intensity was observed using a laser scanning confocal microscope. Scale bar: 50 μm. (**d**) THP-1 cells were treated with 10 mM AAPH for the indicated periods in the absence of H_2_ and then time dependent increase in Ca^2+^-signal was monitored by intracellular Fluo-3 fluorescence intensity as described in (**c**). (**e**) Fluo3-positive cells were semi-quantified after the treatment with 10 mM AAPH for 3 h in the absence or presence of the indicated concentrations of H_2_. **P < 0.01 *vs.* no H_2_ (*n* = 3). (**f**) THP-1 was treated with 10 mM AAPH for 3 h in the presence of the indicated concentrations of H_2_. The translocation of NFAT into the nucleus was examined as described in Methods and shown by immunostaining in yellow. The nucleus was counter-stained with Hoechst 33342 as shown in blue. Scale bar: 50 μm. (**g**) The NFAT-expressing areas were semi-quantified and shown by the ratio of NFAT in the nucleus with that in cytosol. *P = 0.023 and **P < 0.01 *vs*. no H_2_ (*n* = 10). (**h**,**i**) The expressions of the NFAT-target genes (*TNF-α*, *EGR1*, and *ATF3*) (**h**) and non-NFAT target gens (*NFKB2* and *HMOX1*) (**i**) were estimated using RT-PCR coupled with a TaqMan probe (the probes are listed in Supplementary Table 2). The names of the genes are described in Supplementary Table 1. *P = 0.015 (for *ATF3*) (+AAPH and +H_2_ *vs*. +AAPH and –H_2_). #*P* = 0.14 (for *HMOX1*) (+AAPH and –H_2_ *vs*. +AAPH and + H_2_), and **P < 0.01 (*n* = 3)

Autoxidation of unsaturated fatty acids, including PAPC, proceeds by a free radical chain reaction^13^, and·OH is the primary trigger for this reaction^13^,^21^,^22^. We previously showed that H_2_ reduces ·OH levels inside cultured cells by using the spin trapping method and a specific fluorescent indicator^1^. Thus, in this study, we investigated the effects of H_2_ on the lipid free radical chain reaction by using cultured cells. To initiate a free radical chain reaction inside the cells, we used 2,2′-azobis(2-methylpropionamidine)dihydrochloride (AAPH)^23^, which is not affected by H_2_ (Supplementary Fig. 4) and is suitable for the slow generation of free radicals by a spontaneous chemical reaction. The lipid free radical chain reaction results in the production of lipid peroxides (LPOs)^21^,^24^, which can be detected using the fluorescent dye Liperfluo^25^. Thus, we exposed cultured THP-1 cells to AAPH and estimated LPO production based on the Liperfluo signal. The Liperfluo signal significantly decreased in the presence of low levels of H_2_ gas (e.g., 1.3%; Fig. 5a,b). Thus, even at such low concentrations, H_2_ has the potential to reduce the generation of LPOs by suppressing the initiation and/or propagation of free radical chain reactions in cultured cells.

Next, we determined whether the responses induced by chemically produced H_2_OxPAPC (Figs 3 and 4) could simulate the effects induced by the free radicals in cultured cells. When THP-1 cells were exposed to AAPH, the cellular Ca^2+^ levels increased (Fig. 5c) in a time-dependent manner (Fig. 5d), as shown by the analysis of Fluo-3, and the Ca^2+^ signaling was suppressed by H_2_ (Fig. 5c,e). NFAT was also activated, as shown by the translocation of NFAT into the nucleus (Fig. 5f,g), and the nuclear translocation of NFAT were recovered by H_2_ (Fig. 5f,g). Moreover, the free radical inducer stimulated the expression of some target genes of NFAT, including *TNF-α*, early growth response protein 1 (*EGR1*), and activating transcription factor 3 (*ATF3*), which have been shown in Supplementary Table 1, and H_2_ decreased their expressions (Fig. 5h), suggesting that H_2_ regulates these genes via the NFAT pathway.

In contrast, AAPH-mediated activation of CREB was not observed (Supplementary Fig. 5) in this cultured cell line, regardless of the stimulation of cellular Ca^2+^. In particular, the expression of the CREB-target gene *NFKB2* (NF-κB, subunit 2 gene) was not affected by AAPH (Fig. 5i), and the expression of *HMOX1* (Heme Oxygenase 1 gene), a nuclear factor-E2-related factor 2 (Nrf2)-target, was slightly but not significantly increased by H_2_ (Fig. 5i). This result was consistent with those of a previous study^26^. Thus, the NFAT pathway could mainly contribute to the H_2_-dependent transcriptional response induced by free radicals at least in THP-1 cells.

**Figure 6:**
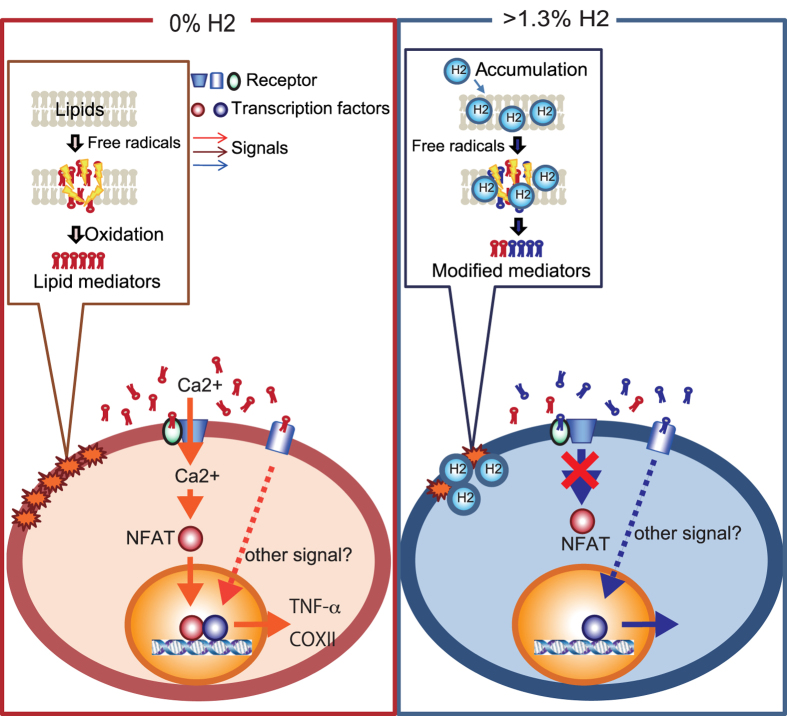
A model of the proposed pathway. When free radical chain oxidation generates oxidized phospholipid mediators, Ca^2+^ signaling is induced, followed by the activation of calcineurin and subsequent induction of the NFAT pathway. On the other hand, H_2_ modifies the production of oxidized phospholipids by modulating free radical chain reactions. The putative oxidized phospholipids appear to function as antagonists and lead to a decline in Ca^2+^ signaling.

Taken together, these cellular responses, at least partly, are in agreement with those obtained using the *in vitro* H_2_-dependent products of OxPAPC species (Figs 3, 4). Therefore, we proposed a model in which H_2_ is linked to the modulation of Ca^2+^ signal transduction and the NFAT pathway via oxidized phospholipid species, as illustrated in Fig. 6.

## Discussion

While the biological effects of H_2_ have been evaluated in more than 300 animal studies and 10 clinical analyses in humans^6^,^7^, the molecular mechanisms by which H_2_ at low concentrations exerts its multiple effects on signal transduction remained unknown. Therefore, in this study, we aimed to examine how H_2_ regulates signal transduction pathways that mediate gene expression. Our results suggested that low concentrations of H_2_ modulated Ca^2+^ signal transduction and regulated gene expression by modifying the production of oxidized phospholipid species. Hence, these data provide important insights into one of the molecular mechanisms by which H_2_ mediates gene expression.

H_2_ can be ingested via several methods. Drinking of H_2_-infused water (H_2_-water) has been shown to be efficacious in the treatment of various diseases in animal models and humans^6^,^7^; however, H_2_ can be infused up to only 0.8 mM under atmospheric pressure, and drinking saturated H_2_-water provides a blood concentration up to only ~10 μM, with a short dwelling time in the body^11^,^27^. Moreover, inhaling 1%–4% (v/v) H_2_ gas was shown to be effective, reaching concentrations of 8–32 μM H_2_ in the blood^1^,^4^,^5^. However, initiation of cellular signals by these low concentrations of H_2_ may be difficult to be explained because H_2_ should be too inert to react with most molecules. To activate H_2_ for reaction with the other molecules, a sufficient level of a putative catalyst must be present; however, it is unlikely that such a putative catalyst would be abundant inside cells. Moreover, H_2_ is very small and is unlikely to bind to a putative H_2_-binding receptor because its intra-molecular fluctuation would be expected to lead to instability in terms of thermodynamics, as previously discussed^28^. Thus, it was unknown how low concentrations of H_2_ regulate signal transduction and gene expression.

Since increased oxidative stress involving ·OH triggers free radical chain reactions, we assumed that the chemically produced mediators derived from phospholipids could contribute to various pathogenic conditions. In the present study, we verified that a small amount of H_2_ (as low as 1.3%) affected free radical-dependent lipid peroxidation, from which oxidized lipid mediators should be derived^22^.

Generally, H_2_ hydrogenates unsaturated fatty acids at higher temperatures with a palladium catalyst. To the best of our knowledge, no studies have examined autoxidation-dependent hydrogenation at approximately 1% (v/v) H_2_ gas at 37 °C without any catalysts. Although H_2_ was thought to be inert in the absence of a catalyst at body temperature, we demonstrated that approximately 1% (v/v) H_2_ suppressed autoxidation of an unsaturated fatty acid in a chemically pure system in this study; thus, our data provided insights into the biological activities of H_2_.

There are two possibilities: the effects of oxidized phospholipid species on Ca^2+^ signaling may be explain by decreased levels of a putative agonist that induces Ca^2+^ signaling or by increased levels of a putative antagonist that disturbs Ca^2+^ signaling. Although we could not identify these species in this study, it is likely that H_2_ modified the production of reduced forms of oxidized phospholipid species during free radical chain reactions by the following previous findings: POVPC is a bioactive phospholipid-mediator that is produced by chemical oxidation of PAPC, and the reduced form of POPVC has been shown to function as an antagonist for signal transduction^18^. Thus, it is possible that during a lipid free radical chain reaction, H_2_ contributes to the generation of a reduced form(s) that function(s) as an antagonist(s). Therefore, we proposed a hypothetic model in which H_2_ is linked to the modulation of Ca^2+^ signal transduction and the NFAT pathway via oxidized phospholipid species as illustrated in Fig. 6.

Previous studies have shown that 1%–4% was efficacious in inhaling H_2_ gas in various animal experiments^1^,^3^,^4^,^29^,^30^,^31^. Since a mixed gas containing 1.3% H_2_, 30% O_2_ and 68.7% N_2_ is available, the effects of around 1.3% needed to be investigated in further studies, including clinical ones^5^. The effective concentrations of H_2_ gas were approximately consistent throughout this study (Figs 2, 3, 4, 5).

No receptors involved in Ca^2+^ signaling were identified in the present study; however, a previous study showed that some chemically oxidized phospholipid mediators, such as 9-HODE and 11-HETE, could bind a G-protein coupled receptor (G2A) to induce Ca^2+^ signaling^17^. Thus, putative oxidized phospholipid mediators or antagonists might bind to G-protein coupled receptors to modulate signal transduction.

In addition to the anti-oxidative roles of H_2_, it has shown to function as an immunosuppressant in allograft transplantation^32^. This immunosuppressant effect can be explained by the suppression of NFAT activation because an immunosuppressant such as CsA and tacrolimus (FK506) acts through the inactivation of calcineurin. Since pro-inflammatory cytokines are regulated by NFAT-dependent mechanisms^20^, the anti-inflammatory effects by H_2_ can be explained by the suppression of NFAT. Additionally, the anti-allergic effects of H_2_ can be explained by the decrease in Ca^2+^/NFAT signaling^33^.

A considerable number of the multiple functions of H_2_, as shown by previous studies, might be explained by the link between H_2_ and NFAT because of the numerous multiple functions of NFAT^20^,^34^. For example, decreased expression of inducible nitric oxide synthase (iNOS) by H_2_^35^ can be explained by the inactivation of NFAT^36^. The suppression of osteoclast differentiation^37^ and improvement of hypertension^38^,^39^ by H_2_ could involve the NFAT pathway^40^,^41^. Moreover, the decreased expression of gene products through an NFAT-dependent pathway might be involved in α-synuclein-induced degeneration of midbrain dopaminergic neurons in Parkinson’s disease^42^. This NFAT-dependent pathway might explain the beneficial effects of H_2_ in these patients^8^. Further studies are needed to elucidate the mechanisms by which H_2_ exerts multiple functions in terms of the involvement of the NFAT pathway.

In summary, in this study, we investigated the link among H_2_, oxidized phospholipids, and Ca^2+^ signaling. Further studies are warranted to identify the H_2_-dependent bioactive mediator(s). Our data provided important insights into one of the mechanisms by which H_2_ regulates signal transduction and gene expression; however, H_2_ might contribute to other types of signaling pathways as well because H_2_ regulates many genes belonging to various signaling pathways. A more detailed understanding of the molecular mechanisms of H_2_-dependent signal transduction and gene expression is expected to facilitate the application of H_2_ in a wide range of medical applications.

## Methods

### Measurement of H_2_

Gases containing H_2_ were prepared by mixing H_2_, O_2_, N_2_, and CO_2_ at various concentrations from each gas cylinder equipped with a flow meter. The H_2_ concentration in the mixed gas or air was tested in each experiment by using gas chromatography (Breath Gas Analyzer, Model TGA2000; TERAMECS Co. Ltd., Kyoto, Japan) as described previously^1^. For the measurement of H_2_ in the solvent, H_2_ was transferred to the air phase in a closed aluminum bag, and the concentration of H_2_ measured by using gas chromatography as described previously^1^. The aluminum used in the bag was covered with a plastic film to avoid any influence of aluminum.

### Autoxidation of linoleic acid-film

Linoleic acid and (±)9-HODE were purchased from Nacalai Tesque (Kyoto, Japan) and CAY (MI, USA), respectively. Linoleic acid was dissolved in cyclohexane to 16 mM, and 2 μL was dispensed into each glass tube (ϕ10 × 50 mm) that had been filled with argon gas; it was allowed to dry up to form a linoleic acid-film at the bottom of a glass tube. The glass tubes were placed into a closed aluminum bag, and the gas in the bag was completely replaced with the indicated mixed gas, where pure H_2_, O_2_, and N_2_ were obtained from separate cylinders. The bag was incubated at 37 °C for 20 h for the autoxidation, and 0.2 mL cyclohexane was immediately added to the glass tube to obtain 0.16 mM peroxidized linoleic acid. The concentration of conjugated diene was estimated by measuring the absorption at 234 nm while scanning from 200 to 300 nm.

### Autoxidation of pure PAPC in air in the absence or presence of H_2_

Chemically synthesized pure PAPC was purchased from Avanti Polar Lipids (Alabaster, AL, USA). PAPC was autoxidized in air as described previously^43^. Briefly, 0.5 mg of PAPC in 50 μL of chloroform was transferred to a ϕ10 × 50 mm glass tube and dried up under a gentle stream of nitrogen. The lipid residue was allowed to autoxidize in air with 100% humidity at 25 °C in the presence or absence of the indicated concentrations of H_2_ gas in a closed aluminum bag for the indicated periods, and then suspended in PBS at a concentration of 0.5 mg/mL.

### Estimation of OxPAPC with Liperfluo

OxPAPC was assayed in ethanol with Liperfluo as described previously^25^. Five min after adding OxPAPC to 1 μM Liperfluo at room temperature, the fluorescence was measured using a fluorescence spectrophotometer (RF-5300PC; Shimadzu Corporation, Kyoto, Japan), where wavelengths of excitation and emission were set at 488 and 535 nm, respectively.

### Measurement of Ca^2+^ signaling

Intracellular Ca^2+^ in THP-1 cells treated with OxPAPC was measured using a Calcium Kit-Fluo 4 (CS22; Dojindo, Kumamoto, Japan) according to the manufacturer’s protocol. Briefly, THP-1 cells were washed with PBS and incubated with 4.5 μM Fluo 4-AM in recording medium (20 mM HEPES, 115 mM NaCl, 5.4 mM KCl, 0.8 mM MgCl_2_, 1.8 mM CaCl_2_, 13.8 mM glucose) containing 0.064% pluronic F-127 and 1.25 mM probenecid for 30 min at 37 °C. The cells were washed with PBS and resuspended in recording medium containing 1.25 mM probenecid. The cells were seeded on 35-mm glass-bottomed dishes and then stimulated with 100 μg/mL OxPAPC or H_2_OxPAPC, followed by 25 μM ATP. The changes in Fluo 4-AM fluorescence were monitored using a laser scanning confocal microscope (FV1200; Olympus Corporation, Tokyo, Japan). The strength of each fluorescent signal in 400 cells was examined and judged as positive if there was greater than 30% of the ATP signal.

Intracellular Ca^2+^ of THP-1 cells treated with the free radical inducer AAPH^23^ was measured by Fluo-3 (F-23915; Molecular Probes, Eugene, OR, USA). Briefly, THP-1 cells were pre-incubated with 2 μM Fluo 3-AM in HBSS containing 0.02% pluronic F-127 for 30 min at 37 °C, resuspended in RPMI1640 (with 10% FBS) containing 2.5 mM probenecid, seeded in 24-well plates, and then treated with AAPH in the presence or absence of H_2_. Changes in Fluo-3 fluorescence signals were observed using a laser scanning confocal microscope (FV1200; Olympus).

### Mass spectrometric analysis and presentation of data using heat maps

OxPAPC (dissolved in chloroform at 2.5 mg/mL) was analyzed using by electrospray ionization-mass spectrometry (ESI-MS) by using an LTQ ORBITRAP XL mass spectrometer (Thermo Fisher Scientific, San Jose, CA, USA) equipped with a nitrogen sheath gas flow rate of 40 AU at 300 °C. The sample was directly infused. The scanning range was from *m/z* 250 to 1000 in the positive ion detection mode. The ion spray voltage was set to 4 kV. OxPAPC species were identified according to their *m/z* values and confirmed using mass spectrometric analysis as described previously^14^,^44^,^45^.

Two independent experiments were performed. The average of the data was used for construction of a heat map and displayed in mass spectrometric profiles. In the heat map, bands were arranged according to molecular mass from small to large, and the strength of each band obtained from H_2_OxPAPC was compared with those by OxPAPC. Red and green bands represented increased and decreased levels as compared with those of OxPAPC, respectively. The mass spectrometric display indicates the average band from two experiments. Only when bands were detected by all of 10 experiments (two experiments at 0%, 0.2%, 0.3%, 1.3% and 5% of H_2_), they were adopted.

### Comprehensive analysis of gene expression

THP-1 cells were exposed for 4 h to PAPC or OxPAPC, H_2_[1.3%]OxPAPC, and H_2_[5%]OxPAPC that had been autoxidized for 3 days with 0%, 1.3%, or 5% H_2_, respectively. Total RNA was extracted using an RNeasy Mini Kit according to the manufacturer’s protocol (Qiagen, Valencia, CA, USA) and labeled using a Low-Input QuickAmp Labeling Kit, One-Color (Agilent Technologies, Santa Clara, CA, USA). Gene expression analysis was performed on samples from three independent experiments using a microarray (SurePrint G3 Human GE 8 × 60 K v2 Microarray; Agilent Technologies). The raw microarray data were deposited in the Gene Expression Omnibus (GEO; accession number, GSE62434; http://www.ncbi.nlm.nih.gov/geo/query/acc.cgi?acc=GSE62434). CREB target genes were selected according to the CREB Target Gene Database (http://natural.salk.edu/CREB/), while NFAT target genes were selected by reference to Medline, as listed in Supplementary Table 1. Signal transduction pathways associated with each gene were identified according to the KEGG Pathway Database (http://www.genome.jp/kegg/pathway.html).

### Quantitative real-time PCR

To quantify mRNA levels, quantitative real-time PCR was carried out using TaqMan Probe and Premix Ex Taq (Probe qPCR; TaKaRa Bio Inc., Shiga, Japan) in a TaKaRa PCR Thermal Cycler Dice TP960 (TaKaRa Bio) according to the manufacturer’s protocols. To normalize mRNA expression levels, glyceraldehyde 3-phosphate dehydrogenase (GAPDH) was used as an endogenous internal control. Primers and probes used for RT-PCR are described in Table 2.

**ELISA (**Enzyme-linked immuno-sorbent assay) HAEC and THP-1 cells were treated with PAPC, OxPAPC or H_2_OxPAPC for 22 h. The IL-8 (HAEC) and TNF-α (THP-1) contents in the culture media were determined using Human CXCL8/IL-8 Quantikine ELISA Kit (R&D Systems, Minneapolis, MN, USA) and Human TNF-α Quantikine ELISA Kit (R&D Systems, Minneapolis, MN, USA), respectively, according to the manufacturer’s protocol.

### Detection of lipid peroxidation in cultured cells

THP-1 cells (1 × 10^5^ cells/mL) were stained with 5 μM Liperfluo^25^ for 30 min and then treated with 10 mM of AAPH^23^ for 4.5 h in the absence or presence of the indicated concentrations of H_2_ gas in a closed vessel. The cells were analyzed using a Cell Lab Quanta flow cytometer (Beckman Coulter, Miami, FL, USA).

### Detection of the translocation of NFAT into the nucleus by immunofluorescence

THP-1 cells (1 × 10^5^ cells/mL) were treated with OxPAPC (0.1 mg/mL), or H_2_[2.5%]OxPAPC (0.1 mg/mL) for 1.5 h, which were used for the Ca^2+^ signaling assay, and then the translocation of NFAT was determined using immunofluorescence as follows. The cells were fixed for 20 min with 10% neutral buffered formalin (3.8% formaldehyde), and then permeabilized with 0.2% Triton X-100 in Tris-buffered saline (TBS-T) for 10 min. After the cells were washed, and blocked with 5% nonfat milk in TBS-T, they were incubated with anti-NFAT1 antibodies (1:100 dilution; 25A10.D6.D2; Abcam, Cambridge, MA, USA) overnight at 4 °C, followed by incubation with Alexa Fluor 488-conjugated anti-mouse antibodies (1:400 dilution; A-11029; Life Technologies, Carlsbad, CA, USA) for 1 h at 25 °C. The cells were counterstained with Hoechst 33342. Immunofluorescence was observed using a laser scanning confocal microscope (FV1200; Olympus).

THP-1 cells (1 × 10^5^ cells/mL) were treated with 10 mM AAPH for 3 h in the absence or presence of indicated concentrations of H_2_, and the NFAT translocation was investigated using immunofluorescence as described above.

### Cell culture

THP-1 cells (ATCC) were cultured in RPMI1640 containing 10% FBS. Human aortic endothelial cells (HAEC) were obtained from Lonza and maintained in endothelial cell growth medium [EBM medium + growth supplements+FCS (Lonza)]. Cells were cultured at 37 °C in a 5% CO_2_ humidified atmosphere and were used for experiments from passage 4 to 8.

### Statistical analysis

Statistical differences between groups were assessed by one-way analysis of variance (ANOVA) with Tukey-Kramer post hoc analysis unless otherwise mentioned. Statistical analyses were performed with IBM SPSS21 software. Results were considered significant at *P* < 0.05. When 0.01 < *P* < 0.05, the actual *P* values were noted. Data are presented as means ± standard deviations.

## Additional Information

**How to cite this article**: Iuchi, K. *et al.* Molecular hydrogen regulates gene expression by modifying the free radical chain reaction-dependent generation of oxidized phospholipid mediators. *Sci. Rep.* **6**, 18971; doi: 10.1038/srep18971 (2016).

## Supplementary Material

Supplementary Information
