# Supplementary material for: Molecular hydrogen regulates gene expression by modifying the free radical chain reaction-dependent generation of oxidized phospholipid mediators
Source: Sci Rep. 2016 Jan 7;6:18971. doi: 10.1038/srep18971 (PMC4704061; doi:10.1038/srep18971)
Supplement: Supplementary Information [file srep18971-s1.pdf]

Supplementary Information

# Molecular hydrogen regulates gene expression by modifying the free radical chain reaction-dependent generation of oxidized phospholipid mediators

**Katsuya Iuchi[1,3], Akemi Imoto[1,3,4], Naomi Kamimura[1,3], Kiyomi Nishimaki[1,3], Takashi Yokota[1], Harumi Ichmiya[1], & Shigeo Ohta[1,2,*]**

[1]*Department of Biochemistry and Cell Biology, Graduate School of Medicine, Nippon Medical School, 1-396 Kosugi-machi, Nakahara-ku, Kawasaki-city, Kanagawa 211-8533, Japan*

[2]*Department of Neuroregenerative Medicine, Juntendo University Graduate School of Medicine, 2-1-1 Hongo, Bunkyo-ku, Tokyo 113-8421, Japan*

[3]*These authors contributed equally to this work.*

[4]*Present address: Department of Clinical Chemistry, School of Allied Health Sciences, Kitasato University, 1-15-1 Kitasato, Minami-ku, Sagamihara-city, Kanagawa 252-0373, Japan*

*Corresponding author: ohta@nms.ac.jp

**Supplementary Figure 1.**

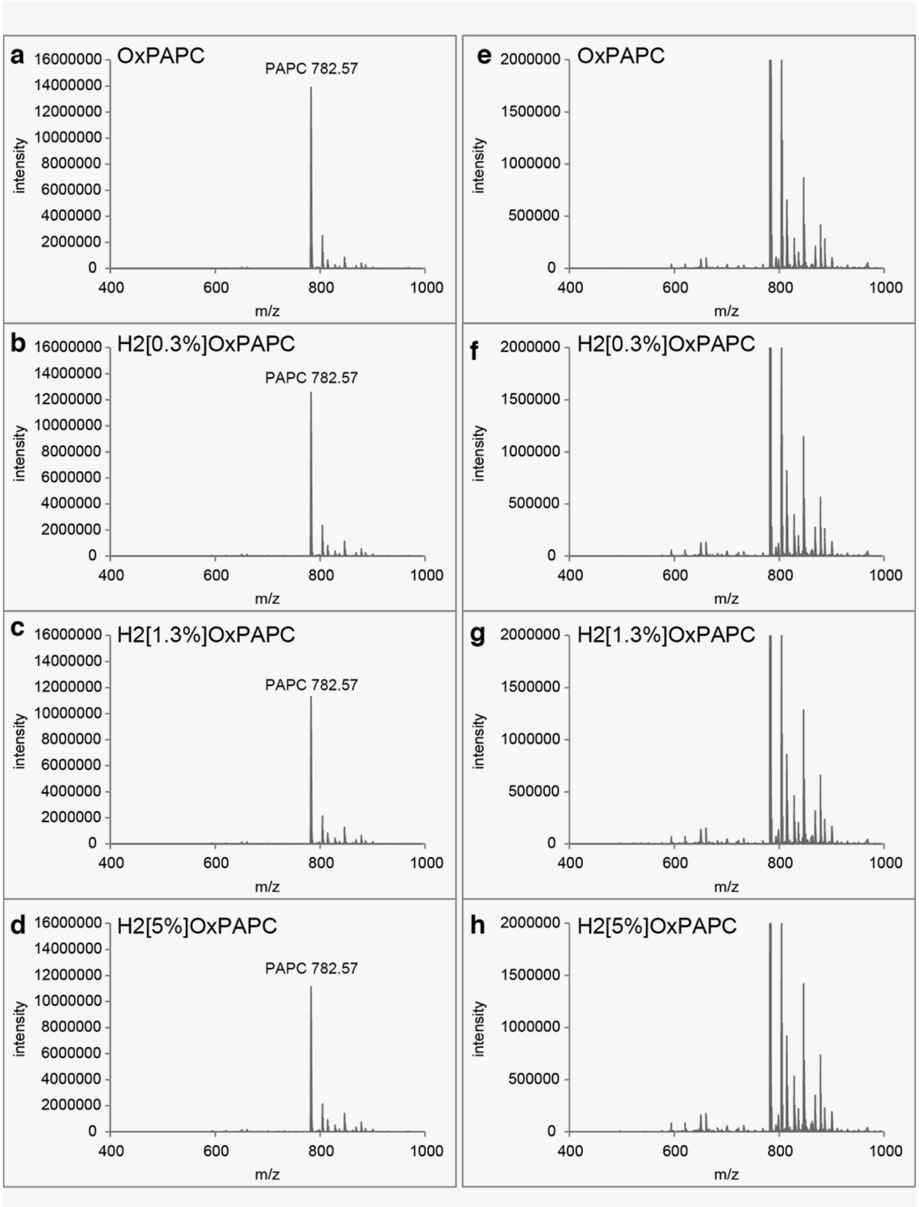


i

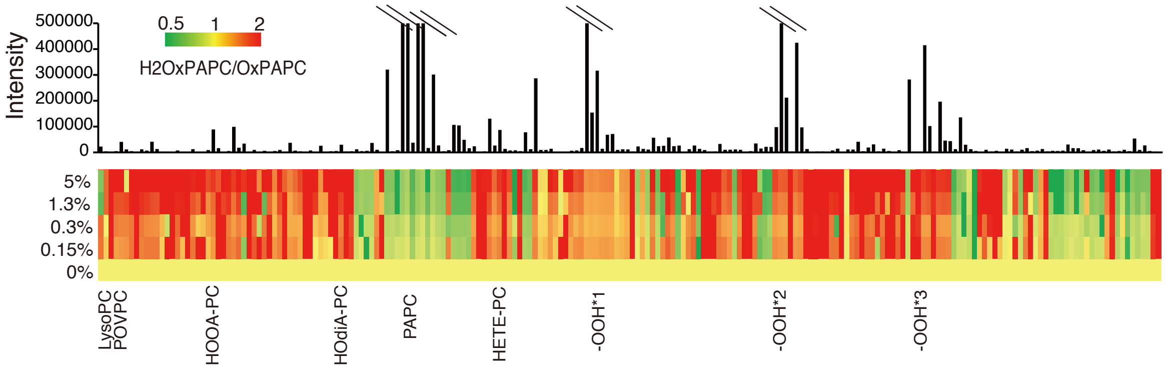

**Supplementary Figure 1. Profiles of mass spectrometry.** PAPC was autoxidized, and subjected to mass spectrometry as described in **Methods**. (**a** and **e**), (**b** and **f**), (**c** and **g**), and (**d** and **h**) indicate the profiles of OxPAPC, $H_2$[0.3%]OxPAPC, $H_2$[1.3%]OxPAPC and $H_2$[5%]OxPAPC, respectively. Vertical and horizontal axes indicate counts, and m/z, respectively. (**e**), (**f**), (**g**), and (**h**) show the expansion in the vertical axes. (**i, upper**)Mass spectrometric profiles show bands corresponding to OxPAPC species. Oxidized PAPCs are identified according to the molecular mass. Lyso, -OOH*1, -OOH*2, and -OOH*3 indicate lysophosphatidylcholines derived from PAPC, mono-peroxide, di-peroxide, and tri-peroxide of PAPC, respectively. (**i, lower**) Each resulting band (the average of two experiments) is shown in a heat map. Bands representing increased and decreased levels ($H_2$OxPAPC *vs.* OxPAPC) are shown as red and green, respectively, according to the color gradient.

**Supplementary Figure 2.**

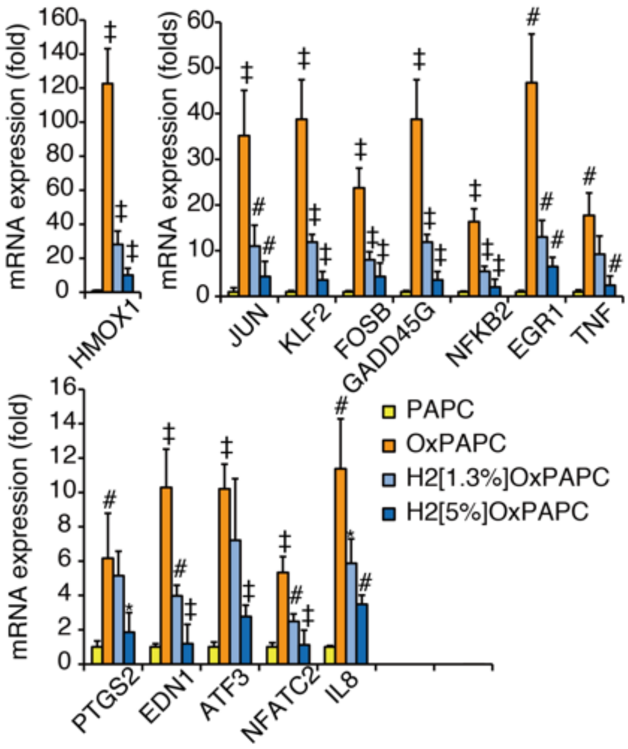


**Supplementary Figure 2. Validation of the expression of the genes selected by microarray analysis by using RT-PCR.** The results of microarray, including marginal genes, were validated by RT-PCR coupled with TaqMan probes. The names of the genes and primer sets are listed in **Supplementary Tables 1** and **2**, respectively. *, #, and ‡ indicate $P = 0.03$, $P < 0.01$, and $P < 0.001$, respectively, for OxPAPC *vs.* PAPC, and $H_2$[1.3%]OxPAPC or $H_2$[5%]OxPAPC vs. OxPAPC.

**Supplementary Figure 3**.

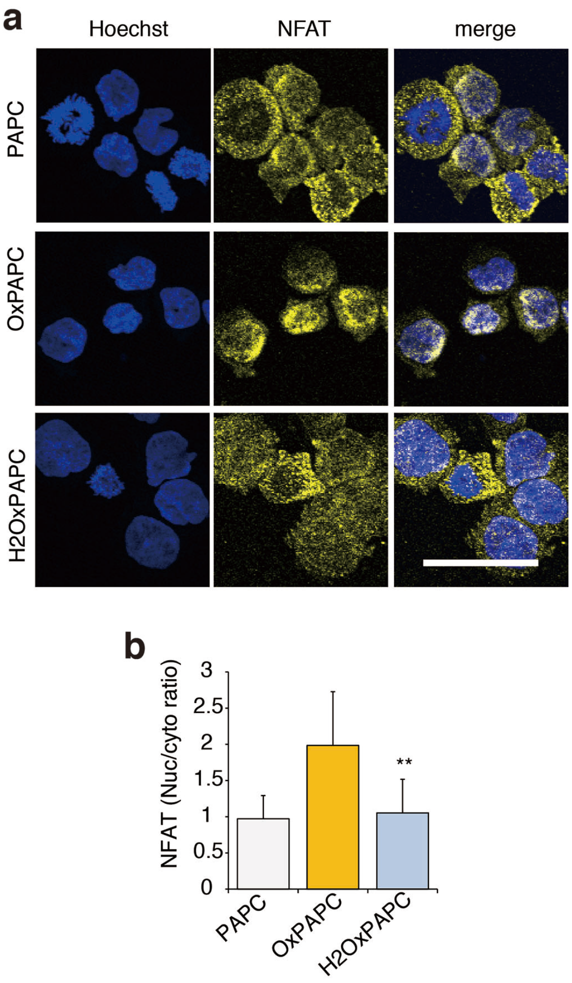


**Supplementary Figure 3**. **Translocation of NFAT to the nucleus is visualized by immunostaining.** OxPAPC and $H_2$OxPAPC were obtained in the absence or presence of 2.5% $H_2$ as performed in **Figure 3**, and then exposed to THP-1 for 1.5 h. (**a**)The cells were immunostained with anti-NFAT1 (yellow) and counterstained with Hoechst 33342 (blue) as described in **Methods.** Scale bar: 50 µm. (**b**)The NFAT-expressing areas were semi-quantified. ** $P < 0.01$ *vs.* OxPAPC ($n = 10$).

**Supplementary Figure 4.**

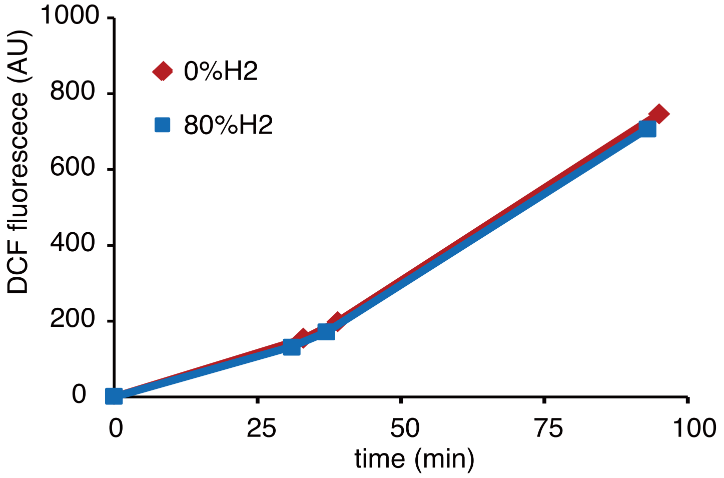


**Supplementary Figure 4. $H_2$ does not react with AAPH** 2',7' dichlorofluorescin diacetate (DCFH-DA) was used for measuring the radical activity of AAPH. Briefly, DCFH was obtained by hydrolyzing DCFH-DA with 0.01 M aqueous NaOH for 30 min at 37°C in the dark. AAPH (1 mM) and DCFH (10 μM) were added to sodium phosphate buffer (0.1 M, pH 7.2) in the absence or presence of 80% $H_2$. The fluorescence intensity of DCFH was measured at 520 nm with excitation at 500 nm.

**Supplementary Figure 5.**

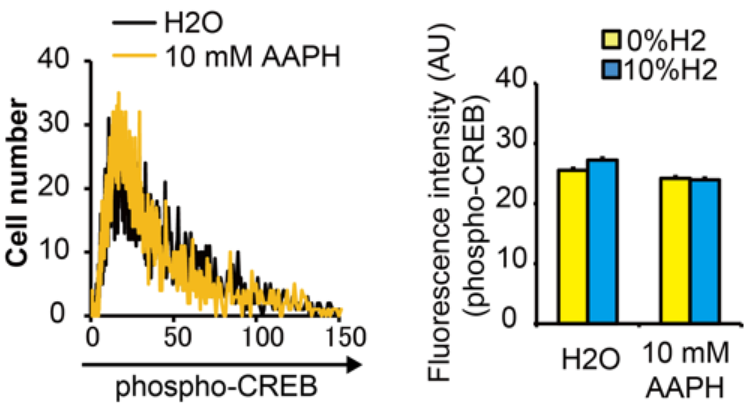


**Supplementary Figure 5. CREB was not activated by a free radical inducer in THP-1.** THP-1 cells ($1 \times 10^5$ cells/mL) were treated with or without 10 mM AAPH for 3 h in the absence or presence of 10% $H_2$. The cells were fixed for 30 min with 10% neutral buffered formalin. After washing, the cells were permeabilized and blocked with 5% bovine serum albumin in Tris buffered saline (pH 7.4) for 30 min. The cells were incubated with anti-phospho-CREB (Ser133) antibodies (1:400 dilution; #9198; Cell Signaling Technology, Danvers, MA, USA) overnight at 4°C, followed by incubation with Alexa Fluor 488-conjugated anti-rabbit antibodies (1:400 dilution; A-11008; Molecular Probes) for 1 h at 25°C. The cells (5,000 cells) were analyzed using flow cytometry using a Cell Lab Quanta flow cytometer (Beckman Coulter, Miami, FL, USA). (**Left**) Representative profiles of flow cytometry in the absence and presence of 10 mM AAPH. (**Right**) Quantification of flow cytometry results.

## Supplementary Table 1 Genes whose expressions were increased by OxPAPC and decreased by $H_2$OxPAPC

| Gene Symbol | Description and Gene accession number | *Expression by PAPC **Increase by OxPAPC (vs. PAPC) **Decrease by H2[1.3%]OxPAPC (vs. OxPAPC) ****Decrease by H2[5%]OxPAPC (vs. OxPAPC) (fold) | | | | Transcription factor and KEGG Pathway (involved in signal transduction) | NFAT target Ref. |
|---|---|---|---|---|---|---|---|
| | | * | ** | *** | **** | | |
| PTGS2 | prostaglandin-endoperoxide synthase 2 (prostaglandin G/H synthase and cyclooxygenase) (PTGS2) [NM_000963] | 40 | 5.7 | 0.25 | 0.34 | NF-kappa B signaling pathway // VEGF signaling pathway // TNF signaling pathway // Retrograde endocannabinoid signaling // Oxytocin signaling pathway | 1,2,3 |
| HMOX1 | heme oxygenase 1 (HMOX1) [NM_002133] | 310 | 110 | 0.25 | 0.17 | HIF-1 signaling pathway // TNF signaling pathway // Melanogenesis | |
| ATP1B2 | ATPase, Na+/K+ transporting, beta 2 polypeptide (ATP1B2) [NM_001678] | 31 | 31 | 0.30 | 0.17 | cGMP-PKG signaling pathway // cAMP signaling pathway //Adrenergic signaling in cardiomyocytes // Thyroid hormone signaling pathway | |
| PLA2G4C | phospholipase A2, group IVC (cytosolic, calcium-independent) (PLA2G4C), [NM_003706] | 185 | 5.6 | 0.31 | 0.25 | MAPK signaling pathway // Ras signaling pathway // VEGF signaling pathway // Fc epsilon RI signaling pathway // GnRH signaling pathway // Oxytocin signaling pathway | |
| SOST | sclerostin (SOST) [NM_025237] | 22 | 13 | 0.34 | 0.09 | Wnt signaling pathway | |
| JUN | jun proto-oncogene (JUN) [NM_002228] | 155 | 15 | 0.34 | 0.27 | Transcription factor// MAPK signaling pathway // ErbB signaling pathway // cAMP signaling pathway // Wnt signaling pathway // Toll-like receptor signaling pathway // T cell receptor signaling pathway // B cell receptor signaling pathway // TNF signaling pathway // Neurotrophin signaling pathway // GnRH signaling pathway // Estrogen signaling pathway // Oxytocin signaling pathway (American trypanosomiasis) | |
| HSPA1A | heat shock 70kDa protein 1A (HSPA1A) [NM_005345] | 2550 | 3.8 | 0.35 | 0.48 | MAPK signaling pathway // Estrogen signaling pathway | |
| PLK2 | polo-like kinase 2 (PLK2) [NM_006622] | 235 | 6.21 | 0.36 | 0.21 | FoxO signaling pathway | |
| KLF2 | Kruppel-like factor 2 (KLF2), [NM_016270] | 3240 | 16 | 0.39 | 0.21 | Transcription // FoxO signaling pathway | 4 |
| HSPA1B | heat shock 70kDa protein 1B (HSPA1B), [NM_005346] | 6710 | 6.9 | 0.40 | 0.30 | MAPK signaling pathway // Estrogen signaling pathway | |
| FOSB | FBJ murine osteosarcoma viral oncogene homolog B (FOSB) [NM_006732] | 132 | 21 | 0.40 | 0.31 | transcription factor | |
| CAMKK1 | calcium/calmodulin-dependent protein kinase kinase 1, alpha (CAMKK1), [NM_032294] | 524 | 3.6 | 0.43 | 0.22 | AMPK signaling pathway // Adipocytokine signaling pathway // Oxytocin signaling pathway | |
| ID3 | inhibitor of DNA binding 3, dominant negative helix-loop-helix protein (ID3), mRNA [NM_002167] | 317 | 9.3 | 0.43 | 0.44 | TGF-beta signaling pathway | 5 |
| JUND | jun D proto-oncogene (JUND), [NM_001286968] | 1495 | 4.2 | 0.45 | 0.25 | Transcription factor// MAPK signaling pathway // Osteoclast differentiation | |
| SOCS1 | suppressor of cytokine signaling 1 (SOCS1)[NM_003745] | 350 | 4.0 | 0.46 | 0.31 | Jak-STAT signaling pathway // Insulin signaling pathway // Prolactin signaling pathway | 6 |
| JUNB | jun B proto-oncogene (JUNB), mRNA [NM_002229] | 157 | 6.1 | 0.47 | 0.36 | Transcription factor // TNF signaling pathway | |
| SGK1 | serum/glucocorticoid regulated kinase 1 (SGK1) [NM_005627] | 159 | 10 | 0.48 | 0.35 | FoxO signaling pathway // PI3K-Akt signaling pathway | |
| FIGF | c-fos induced growth factor (vascular endothelial growth factor D) (FIGF), mRNA [NM_004469] | 27 | 72 | 0.51 | 0.29 | Ras signaling pathway // Rap1 signaling pathway // Cytokine-cytokine receptor interaction // PI3K-Akt signaling pathway // Focal adhesion // Pathways in cancer | |
| GADD45G | growth arrest and DNA-damage-inducible, gamma (GADD45G), [NM_006705] | 113 | 11 | 0.52 | 0.39 | MAPK signaling pathway // FoxO signaling pathway // Cell cycle // p53 signaling pathway | 7 |
| FGF3 | fibroblast growth factor 3 (FGF3), mRNA [NM_005247] | 274 | 15 | 0.54 | 0.26 | MAPK signaling pathway // Ras signaling pathway // Rap1 signaling pathway // PI3K-Akt signaling pathway | 8 |
| RELB | v-rel avian reticuloendotheliosis viral oncogene homolog B (RELB) [NM_006509] | 16276 | 4.3 | 0.54 | 0.37 | MAPK signaling pathway // NF-kappa B signaling pathway | |
| CTSK | cathepsin K (CTSK), [NM_000396] | 5560 | 3.3 | 0.56 | 0.35 | Toll-like receptor signaling pathway | |
| ATF3 | activating transcription factor 3 (ATF3) [NM_001674] | 215 | 7.6 | 0.56 | 0.27 | Transcription factor | 9 |
| BBC3 | BCL2 binding component 3 (BBC3) [NM_014417] | 9388 | 3.1 | 0.59 | 0.42 | p53 signaling pathway // Hippo signaling pathway | |
| RASA4B | RAS p21 protein activator 4B (RASA4B) [NM_001277335] | 557 | 2.8 | 0.59 | 0.31 | Ras signaling pathway | |
| DGKA | diacylglycerol kinase, alpha 80kDa [Source:HGNC Symbol;Acc:2849] [ENST00000546995] | 148 | 7.3 | 0.60 | 0.45 | Phosphatidylinositol signaling system | |

| | | | | | | | |
|---|---|---|---|---|---|---|---|
| CYP27A1 | cytochrome P450, family 27, subfamily A, polypeptide 1 (CYP27A1) [NM_000784] | 668 | 2.9 | 0.60 | 0.51 | PPAR signaling pathway | |
| ATP2B4 | ATPase, Ca++ transporting, plasma membrane 4 (ATP2B4), [NM_001684] | 215 | 2.6 | 0.57 | 0.46 | Calcium signaling pathway // cGMP-PKG signaling pathway // cAMP signaling pathway // Adrenergic signaling in cardiomyocytes | 10 |
| NFKB2 | nuclear factor of kappa light polypeptide gene enhancer in B-cells 2 (p49/p100) (NFKB2) [NM_001261403] | 205 | 3.7 | 0.61 | 0.38 | MAPK signaling pathway // NF-kappa B signaling pathway | |
| NFATC2 | nuclear factor of activated T-cells, cytoplasmic, calcineurin-dependent 2 (NFATC2) [NM_173091] | 386 | 3.6 | 0.61 | 0.25 | Transcription factor// cGMP-PKG signaling pathway // Wnt signaling pathway // VEGF signaling pathway // T cell receptor signaling pathway // B cell receptor signaling pathway // Oxytocin signaling pathway | |
| ITGA10 | integrin, alpha 10 (ITGA10), mRNA [NM_003637] | 254 | 3.3 | 0.62 | 0.32 | PI3K-Akt signaling pathway | |
| CD86 | CD86 molecule (CD86), transcript variant 2, mRNA [NM_006889] | 4229 | 5.5 | 0.64 | 0.11 | Toll-like receptor signaling pathway | |
| RASGRP 2 | RAS guanyl releasing protein 2 (calcium and DAG-regulated) [Source:HGNC Symbol;Acc:9879] [ENST00000377494] | 678 | 2.7 | 0.67 | 0.34 | MAPK signaling pathway // Ras signaling pathway // Rap1 signaling pathway // Chemokine signaling pathway // Platelet activation | |
| EDN1 | endothelin 1 (EDN1) [NM_001955] | 88 | 5.1 | 0.53 | 0.25 | HIF-1 signaling pathway // TNF signaling pathway | 3,11, 12,13 |
| PMAIP1 | phorbol-12-myristate-13-acetate-induced protein 1 (PMAIP1) [NM_021127] | 7459 | 9.1 | 0.69 | 0.39 | p53 signaling pathway | |
| PPARG | peroxisome proliferator-activated receptor gamma (PPARG), [NM_138711] | 457 | 3.0 | 0.71 | 0.38 | Transcription factor// PPAR signaling pathway // AMPK signaling pathway | |
| CPT1B | carnitine palmitoyltransferase 1B (muscle) (CPT1B), [NM_152246] | 4652 | 3.1 | 0.71 | 0.36 | PPAR signaling pathway // AMPK signaling pathway | |
| PPP1R12 B | protein phosphatase 1, regulatory subunit 12B (PPP1R12B), [NM_002481] | 431 | 3.1 | 0.73 | 0.19 | Oxytocin signaling pathway | |
| FLNA | filamin A, alpha (FLNA), transcript variant 1, mRNA [NM_001456] | 386 | 2.5 | 0.74 | 0.32 | MAPK signaling pathway | 14 |
| FBXO32 | F-box protein 32 (FBXO32), transcript variant 1, mRNA [NM_058229] | 501 | 3.3 | 0.74 | 0.49 | FoxO signaling pathway | |
| MAPK8I P3 | mitogen-activated protein kinase 8 interacting protein 3 (MAPK8IP3), [NM_015133] | 3580 0 | 2.6 | 0.75 | 0.41 | MAPK signaling pathway | |
| EGR1 | early growth response 1 (EGR1), [NM_001964] | 2210 | 5.2 | 0.84 | 0.58 | Transcription factor | 15 |
| TNF | tumor necrosis factor (TNF),[NM_000594] | 2850 | 5.7 | 0.95 | 0.60 | MAPK signaling pathway // NF-kappa B signaling pathway // mTOR signaling pathway // Apoptosis // TGF-beta signaling pathway // Toll-like receptor signaling pathway // NOD-like receptor signaling pathway // RIG-I-like receptor signaling pathway // Fc epsilon RI signaling pathway // TNF signaling pathway // Adipocytokine signaling pathway | 16,17 18,19 20 |
| IL8 | interleukin 8 (IL8) [NM_000584] | 91 | 2.3 | PCR | PCR | Chemokine signaling pathway // NF-kappa B signaling pathway // Toll-like receptor signaling pathway // NOD-like receptor signaling pathway // RIG-I-like receptor signaling pathway | 16,21 22,23 |

Gene expression was examined by comprehensive microarray analysis as described in **Methods** (SurePrint G3 Human GE 8x60K v2 Microarray, Agilent Technologies, Santa Clara, CA, USA). Genes that OxPAPC up-regulated (more than 2.5-fold *vs.* PAPC) and that $H_2$[1.3%]OxPAPC and $H_2$[5%]OxPAPC down-regulated (less than 0.75-fold and 0.5-fold, respectively, *vs.* OxPAPC) were selected. When the difference obtained from three tests was not significant ($P > 0.05$), the genes were excluded. Functionally important genes that marginally unfitted the above criteria were added to this table after the validation by semi-quantitative PCR analysis. Transcription factors and target genes to NFAT are listed according to the literature. The contribution to signal transduction was according to the KEGG Pathway Database (http://www.genome.jp/kegg/pathway.html).

In the first line of the table; (*) expression levels treated with PAPC; (**)increase (fold) vs. PAPC; (***)decrease (fold) by $H_2$[0.3%]OxPAPC vs. OxPAPC and (****), and decrease (fold) by $H_2$[5%]OxPAPC vs. OxPAPC.

**References cited in Supplementary Table 1 suggesting the NFAT targets**

1 Mena, M. P. *et al.* NFAT2 regulates COX-2 expression and modulates the integrin repertoire in endothelial cells at the crossroads of angiogenesis and inflammation. *Exp. Cell Res.* **324**, 124-136 (2014).

2 Canellada, A., Ramirez, B. G., Minami, T., Redondo, J. M. & Cano, E. Calcium/calcineurin signaling in primary cortical astrocyte cultures: Rcan1-4 and cyclooxygenase-2 as NFAT target genes. *Glia* **56**, 709-722 (2008).

3 Sugimoto, T. *et al.* Endothelin-1 induces cyclooxygenase-2 expression via nuclear factor of activated T-cell transcription factor in glomerular mesangial cells. *J. Am. Soc. Nephrol.: JASN* **12**, 1359-1368 (2001).

4 Suehiro, J. I. *et al.* Genome-wide approaches reveal functional vascular endothelial growth factor (VEGF)-inducible nuclear factor of activated T cells (NFAT) c1 binding to angiogenesis-related genes in endothelium. *J. Biol. Chem.,* **289**, 29044-29059 (2014).

5 Koltsova, E. K. *et al.* Early Growth Response 1 and NF-ATc1 Act in Concert to Promote Thymocyte Development beyond the Selection Checkpoint. *J. Immunol.,* **179**, 4694-4703 (2007).

6 Okugawa, S. *et al.* Bacterial flagellin inhibits T cell receptor-mediated activation of T cells by inducing suppressor of cytokine signalling-1 (SOCS-1). *Cell. Microbiol.,* **8**, 1571-1580 (2006).

7 Hsiao, H. W. *et al.* Deltex1 is a target of the transcription factor NFAT that promotes T cell anergy. *Immunity* **31**, 72-83 (2009).

8 Holzmann, K. *et al.* Genomic DNA-chip hybridization reveals a higher incidence of genomic amplifications in pancreatic cancer than conventional comparative genomic hybridization and leads to the identification of novel candidate genes. *Cancer Res.,* **64**, 4428-4433 (2004).

9 Dziunycz, P. J. *et al.* The oncogene ATF3 is potentiated by cyclosporine A and ultraviolet light A. *J. Invest. dermatol.,* **134**, 1998-2004 (2014).

10 Kosiorek, M., Podszywalow-Bartnicka, P., Zylinska, L. & Pikula, S. NFAT1 and NFAT3 cooperate with HDAC4 during regulation of alternative splicing of PMCA isoforms in PC12 cells. *PloS one* **9**, e99118 (2014).

11 Strait, K. A., Stricklett, P. K., Kohan, R. M. & Kohan, D. E. Identification of two nuclear factor of activated T-cells (NFAT)-response elements in the 5'-upstream regulatory region of the ET-1 promoter. *J. Biol. Chem.,* **285**, 28520-28528 (2010).

12 Van Sant, C. *et al.* Endothelin signaling in osteoblasts: global genome view and implication of the calcineurin/NFAT pathway. *Mol. Cancer Ther* **6**, 253-261 (2007).

13 Kawamura, T. *et al.* Endothelin-1-dependent nuclear factor of activated T lymphocyte signaling associates with transcriptional coactivator p300 in the activation of the B cell leukemia-2 promoter in cardiac myocytes. *Cir. Res.,* **94**, 1492-1499 (2004).

14 Hayashi, K. & Altman, A. Filamin A Is Required for T Cell Activation Mediated by Protein Kinase C. *J. Immunol.,* **177**, 1721-1728 (2006).

15 Susilowati, H. *et al.* Intermedilysin induces EGR-1 expression through calcineurin/NFAT pathway in human cholangiocellular carcinoma cells. *Biochem. Biophys. Res. Commu.* **404**, 57-61 (2011).

16 Robbs, B. K., Lucena, P. I. & Viola, J. P. The transcription factor NFAT1 induces apoptosis through cooperation with Ras/Raf/MEK/ERK pathway and upregulation of TNF-alpha expression. *Biochim. Biophys. Acta* **1833**, 2016-2028 (2013).

17 Rao, A., Luo, C. & Hogan, P. G. Transcription factors of the NFAT family: regulation and function. *Annu. Rev. Immunol.,* **15**, 707-747 (1997).

18 Goldfeld, A. E., McCaffrey, P. G., Strominger, J. L. & Rao, A. Identification of a novel cyclosporin-sensitive element in the human tumor necrosis factor alpha gene promoter. *J. Exp. Med.,* **178**, 1365-1379 (1993).

19 McCaffrey, P. G., Goldfeld, A. E. & Rao, A. The role of NFATp in cyclosporin A-sensitive tumor necrosis factor-alpha gene transcription. *J. Biol. Chem.,* **269**, 30445-30450 (1994).

20 Oum, J. H. *et al.* Molecular mechanism of NFAT family proteins for differential regulation of the IL-2 and TNF-alpha promoters. *Mol. Cells* **13**, 77-84 (2002).

21 Bochkov, V. N. *et al.* Generation and biological activities of oxidized phospholipids. *Antioxidant. Redox Sig.* **12**, 1009-1059 (2010).

22 Maldonado-Perez, D. *et al.* Prokineticin 1 modulates IL-8 expression via the calcineurin/NFAT signaling pathway. *Biochim. Phys. Acta* **1793**, 1315-1324 (2009).

23 Okamoto, S. *et al.* The interleukin-8 AP-1 and kappa B-like sites are genetic end targets of FK506-sensitive pathway accompanied by calcium mobilization. *J. Biol. Chem.,* **269**, 8582-8589 (1994).

## Supplementary Table 2 Nucleotide sequences of primer sets and TaqMan probes

| Gene | Forward primer | Reverse primer | TaqMan probe |
|---|---|---|---|
| Human | | | |
| HMOX1 | CCACCAAGTTCAAGCAGCTC | CTGACTGCGGGAGTCATCTC | ACCGCTCCCGCATGAACTCCC |
| JUN | CAAGAACGTGACAGATGAGCAG | AGCGTGTTCTGGCTGTGC | CACGAAGCCCTCGGCGAAGCCC |
| KLF2 | AAGTGGCATCTTCTCTCCCA | AATGCCGCAGACAGTACAAA | CAAGGCATCACAAGCCTCGATCC |
| FOSB | GTGAGTGTGTGAGCGCTTCT | AGACAGGCTGAGCCAGAGTT | CACGTTGGCCCTCAAAGCGA |
| GADD45G | GCGAGAACGACATCGACATA | TTCGAAATGAGGATGCAGTG | ACGATAGCCGCCAGCCGCT |
| NFKB2 | TGATAGCGACTCGGACTCTG | GAGCAGCATTTAGCAGCAAG | ACACCCGAAGCAGCTTCCGG |
| EGR1 | GAGATGGCCATAGGAGAGGA | AAGTGGGCAGAAAGGATTGT | TGGCTCTGAGAACCTCCATCTGACC |
| TNF | TCTTCTCGAACCCCGAGTGA | CCTCTGATGGCACCACCAG | TAGCCCATGTTGTAGCAAACCCTCAAGCT |
| PTGS2 | GCTCAAACATGATGTTTGCATTC | GCTGGCCCTCGCTTATGA | TGCCCAGCACTTCACGCATCAGTT |
| EDN1 | ACATTGGTGACAGACCTTCG | GTCAGGAACCAGCAGAGGAT | CCCTGTGGCCGACTCTGCAC |
| ATF3 | GCCGAAACAAGAAGAAGGAG | GCTCCTCAATCTGAGCCTTC | CCGACTCTTTCTGCAGGCACTCC |
| NFATC2 | AATGGAATGGATGCAAATGA | TGCTAAGCTTGTCCCAACAG | AGCCTCGGTCTGCTCAGGCC |
| IL8 | GCCAACACAGAAATTATTGTAAAGCTT | AATTCTCAGCCCTCTTCAAAAACTT | AGAGCTCTGTCTGGACCCCAAGGAAAAC |
| GAPDH | GGGAAGGTGAAGGTCGGA | GCAGCCCTGGTGACCAG | CAACGGATTTGGTCGTATTGGGCG |
| Mouse | | | |
| EGR1 | GAGGAGATGATGCTGCTGAG | TGCTGCTGCTGCTATTACC | AGTTCCTCGGTGCTGCCGGAA |
| ATF3 | CAACAGAGGATGGACGACAC | TGTAGCCAAGGACAAAGCTG | TGCCCAGGGTTCTTCCTCTGC |
| PTGS2 | AGAACCTGCAGTTTGCTGTG | GCTCCTGCTTGAGTATGTCG | TCTGTTGTGCTCCCGAAGCCA |
| TNF | TCGTAGCAAACCACCAAGTG | TTGAGATCCATGCCGTTG | AGGAGCAGCTGGAGTGGCTGAGC |
| GAPDH | CATCACTGCCACCCAGAAGA | ATGTTCTGGGCAGCC | TGGATGGCCCCTCTGGAAAGCTG |
